# Modelling the excess mortality associated with heat waves in Hong Kong: 2014-2023

**DOI:** 10.1371/journal.pone.0345222

**Published:** 2026-11-13

**Authors:** Zhenyuan Liu, Chao Ren, Jingwen Liu, Yurika Kawasaki, David Makram Bishai

**Affiliations:** 1 School of Public Health, The University of Hong Kong, Hong Kong SAR, China; 2 Faculty of Architecture, The University of Hong Kong, Hong Kong SAR, China; 3 School of Public Health, Faculty of Health and Medical Sciences, The University of Adelaide, Adelaide, Australia; Changan University: Chang’an University, CHINA

## Abstract

**Background:**

Heat waves have become increasingly frequent, and prior studies have linked them to elevated mortality risks in Hong Kong. However, estimates of the total excess mortality burden attributable to heat waves in Hong Kong remain limited. This study aimed to estimate excess mortality associated with heat waves in Hong Kong from 2014 to 2023.

**Methods and findings:**

We obtained daily age- and sex-specific mortality rates and population data from the Hong Kong Life Tables and the Census and Statistics Department. Historical temperature data were sourced from the Hong Kong Observatory, and relative risk estimates were derived from published local studies. Using a Monte Carlo approach, we estimated heat-attributable deaths under multiple heat wave definitions and calculated both cumulative excess death counts and age-standardized annual excess death rates per 100,000 population.

From 2014 to 2023, the estimated cumulative number of excess deaths attributable to heat exposure in Hong Kong ranged from 1,455 (95% CI: 1,098–1,812) to 3,238 (95% CI: 3,234–3,242), depending on the heat wave definition applied. In 2023, the annualized excess death rate ranged from 2.95 per 100,000 population (95% CI: 2.41–3.50) to 5.09 per 100,000 population (95% CI: 5.07–5.12). Variations in heat wave frequency and intensity were associated with corresponding fluctuations in mortality burden. Excess mortality was disproportionately higher among males and adults aged 65 years or above, indicating greater vulnerability in these groups.

**Conclusions:**

Between 2014 and 2023, extreme heat was associated with an estimated 1,455–3,238 excess deaths in Hong Kong. If considered as an independent health condition, the mortality burden attributable to extreme heat would rank among the leading causes of death in Hong Kong, with annual mortality rates comparable to those of diabetes. Older adults bore a substantially greater mortality burden than younger and working-age populations. These findings underscore the importance of evidence-based heat-health policies and targeted interventions to protect vulnerable populations, particularly older adults, during periods of extreme heat.

## Introduction

The intensification of climate change in recent years has resulted in more frequent heatwaves [1]. According to a recent World Meteorological report, there is an 80 percent likelihood that the annual average global temperature will temporarily exceed 1.5°C above pre-industrial levels for at least one of the next five years [2], positioning extreme heat as one of the most pressing and rapidly escalating public health threats of our time. Physiologically, extreme heat overwhelms the body’s thermoregulatory capacity, precipitating a cascade of adverse outcomes ranging from heat exhaustion and heatstroke to the exacerbation of cardiovascular, respiratory, and renal conditions, ultimately elevating mortality risk—a pattern well-documented across diverse populations and climatic settings [3–5], These risks are compounded in densely populated cities characterised by ageing urban infrastructure, poorly insulated buildings, and inadequate ventilation, where urban heat island effects further amplify indoor thermal exposure during heat wave events [6,7].

A substantial body of epidemiological literature has established the temperature-mortality relationship using daily time series analyses, quantifying the increased probability of death on or after a hot day through relative risk estimates [8–11], attributable fractions [9], and excess mortality rate [12,13]. Notwithstanding these advances, relative risk metrics are inherently limited in their capacity to convey the absolute public health burden of extreme heat to policymakers, clinicians, and the general public [14]. Some studies have reported the number of heat-attributable deaths [9,15], but attributing deaths solely to heat remains methodologically challenging and prone to underestimation, as heat exposure frequently acts as a precipitating factor that accelerates death from pre-existing conditions rather than serving as the sole recorded cause [16]. Excess death estimation, which is defined as the difference between observed and expected deaths under non-heat-wave conditions, addresses such limitation by capturing the full spectrum of heat-potentiated mortality, irrespective of the cause recorded on the death certificate. This approach also mitigates ascertainment bias inherent in cause-of-death records that systematically fail to attribute heat as a contributing factors [3,17,18].

Despite a growing body of evidence characterising the temperature-mortality relationship in Hong Kong through relative risk estimation [8–11], the field has yet to take the critical next step of translating these estimates into absolute excess death counts or age-standardised excess death rates. This omission is consequential: absolute metrics occupy the same measurement space as official cause-of-death statistics, enabling direct comparison of heat-attributable mortality with that of recognised chronic diseases and rendering the burden of extreme heat legible within existing public health surveillance and priority-setting frameworks. Furthermore, no prior study has systematically examined how the choice of heat wave definition affects absolute mortality burden estimates in Hong Kong, nor has any study propagated definitional and statistical uncertainty within a unified simulation framework. To address these gaps, this study estimated, for the first time, the absolute number of excess heat-related deaths and the corresponding age-standardized excess death rates in Hong Kong from 2014 to 2023, applying four locally validated heat wave definitions within the analytic simulation framework. By situating heat-attributable mortality within the same metric space as conventional cause-of-death reporting, this study provides a novel quantitative foundation for recognizing extreme heat as a formally measurable and policy-relevant contributor to premature mortality in Hong Kong.

## Method

### Definition of heat waves

Given the absence of a universally accepted definition for a heat wave, we employed four distinct definitions that capture heat wave conditions in Hong Kong, as delineated in previous local studies (Table 1). The first definition identifies a heat wave as a 20- day period commencing when the daily mean temperature exceeds the 99th percentile threshold of 30.60 °C. This duration accounts for the lagged effects of a single hot day, which may persist for up to 20 days, as estimated by Liu et al. (2020) [9]. Additionally, to qualify as an independent heat wave event, the initial day must be separated by at least 20 days from the preceding day that surpassed the threshold. The second definition characterizes a heat wave based on the number of consecutive days with daily maximum temperatures exceeding 33 °C. The third definition focuses on the number of consecutive days with daily minimum temperatures above 28 °C. Lastly, the fourth definition determines a heat wave by the simultaneous occurrence of hot days and hot nights, following the metrics established by Wang et al. (2019) [11]. These diverse criteria were selected for our analysis based on their well-established associations with increased excess mortality risks, while controlling for lagged effects and common confounding factors such as humidity, air pollutants, and seasonality.

**Table 1:** Heat wave definitions used as the basis for the present analysis.

Type	Name	Heat Wave Criteria	Estimation of Relative risks (RRs)	Source
HWD_Tavg	Daily average temperature-based definition	• A consecutive series of 20 days beginning with a day that daily mean temperature≥30.60°C; • An independent heat wave is classified when the first day is at least 20 days apart from the last day exceeding the threshold.	• RRs were calculated on a daily basis. • For each independent heat wave, RRs were assessed across multiple lag periods, divided into three distinct phases: • 0th day: Captures the immediate effects of the heat wave. • 1st to 5th days: Addresses delayed effects following the heat wave. • 6th to 21st days: Evaluates long- term effects associated with the heat wave.	Liu et al. (2020) [9]
HWD_Tmax	Daily maximum temperature–based definition	• A single day when the daily minimum temperature ≥28°C is considered as a very hot day (VHD) • A consecutive series of VHNs with lengths longer than 5 days (5HD).	• RRs were calculated on a daily basis. •No detailed statistics are provided on the lagged effects of heat waves following the 5HD pattern	Wang et al. (2019) [11]
HWD_Tmin	Daily minimum temperature–based definition	• A single day when the daily minimum temperature ≥28°C is considered as a very hot night (VHN) • A consecutive series of VHNs with lengths longer than 5 nights (5HN).	• RRs were calculated on a daily basis. • No detailed statistics are provided on the lagged effects of heat waves following the 5HN pattern	
HWD_Tcombined	Combined daily temperature threshold-based definition	• Defined as two consecutive days with maximum temperatures of 33°C or higher and three consecutive nights with minimum temperatures of 28°C or higher (2D3N). • The mortality effects from 2D3N heat waves show a significant five-day lag after their onset.	• RRs were calculated on a daily basis. • For each independent heat wave, RRs were assessed across multiple lag periods, divided into two distinct phases: • Days observed as part of 2D3N event • Subsequent five days following this event	

### Data source

All data used in this analysis were obtained from official government and institutional sources with established quality assurance frameworks. The analytical framework proceeded in three steps as described below.

First, daily temperature records from the Hong Kong Observatory (HKO) station were used to construct heat wave scenarios over the study period from 2014 to 2023 [19]. The HKO operates under the standards of the World Meteorological Organization (WMO), applying systematic instrument calibration, automated range-check algorithms, and cross-station consistency checks prior to public release, ensuring measurement accuracy and temporal reliability.

Second, mortality rates and life table data sourced from the Hong Kong Census and Statistics Department (CSD) were used to construct the “business-as-usual” baseline scenarios representing expected daily death counts in the absence of any heat wave shock [20–22]. The CSD compiles official demographic and mortality statistics through a legally mandated civil registration system under the Births and Deaths Registration Ordinance (Cap. 174), in which all registered deaths undergo cause-of-death verification by certified medical practitioners before datasets are subjected to rigorous internal validation. Population estimates, also obtained from the CSD, follow internationally recognised demographic methodologies endorsed by the United Nations Statistics Division and are further verified through post-enumeration surveys and reconciliation checks. The time series of expected death counts was generated by multiplying age- and sex-specific death rates from the Hong Kong Life Tables by the corresponding CSD population figures, with internal consistency between these two inputs ensured by their derivation from the same validated institutional source.

Third, the relative risks (RRs) estimated by previously published local studies, as shown in Table 1 and S1 Table, were applied to the baseline mortality counts to estimate excess heat-related deaths under each of the four heat wave scenarios. The use of locally derived relative risk estimates ensures that the epidemiological parameters reflect the specific climatic, demographic, and behavioural context of Hong Kong. All datasets employed in this analysis are publicly available and have been independently used and validated across numerous peer-reviewed epidemiological studies, lending additional external corroboration to their authenticity and accuracy.

### Transformation of relative risks

We used estimates of the RRs due to heat from Liu et al. (2020) [9] and Wang et al. (2019) [11]. The theoretical basis for EHD involves a counterfactual comparison, contrasting observed conditions (e.g., heat exposure) with a hypothetical reference state (e.g., normal conditions without heat exposure). RR represents the ratio of the probability of death under heat exposure to that in a reference group under ordinary temperatures, as a key variable in calculating EHDs. The RRs published by Liu et al. (2020) [9] and Wang et al. (2019) [11] were estimated separately for age groups and for sex, but not for all age-by-sex cells simultaneously. To obtain age-sex-specific RRs, we employed a log-additive decomposition based on the assumption that age and sex effects on heat-related mortality risk operate independently on the multiplicative (relative risk) scale—a standard assumption in log-linear Poisson regression models for count data. Under this assumption, the joint relative risk for an individual of sex i and age group j is modelled as:


$$\begin{array}{c} ln\left({RR}_{age=j,sex=i}\right)=ln\left({RR}_{age=j}\right)+ln\left({RR}_{sex=i}\right)-ln({RR}_{all}) \end{array}$$ (1)

The term $-ln(RR_{all})$ is subtracted to prevent double-counting the average population-level risk already embedded in each of the marginal estimates $RR_{age=j}$ and $RR_{sex=i}$. Equivalently, this can be written as:


$$\begin{array}{c} RR_{age=j,sex=i}=\;\frac{RR_{age=j}\times RR_{sex=i}}{RR_{all}} \end{array}$$ (2)

This approach is analogous to methods used in health economic decision modelling for combining evidence from multiple sources when joint estimates are unavailable [23]. The assumption of multiplicative independence between age and sex effects is a widely used simplifying assumption in the absence of fully stratified data, and its validity is supported by the general epidemiological observation that older age and male sex each independently elevate heat mortality risk. Detailed adjusted RRs are provided in S1 Table.

Adjusted RRs and their corresponding standard deviations are also logarithmically transformed. This transformation is essential as the RRs represent a ratio, and confidence intervals for their parameters are commonly estimated on the logarithmic scale, indicating a log-normal distribution [23]. Consequently, we generated 1000 values of log-normally distributed RRs, using a pseudo-random number generator in Stata by using Equation 3 as follows:


$$\begin{array}{c} RR=exp((invnorm\;(uniform(rand)\times logSD+logRR)) \end{array}$$ (3)

### Estimation of excess mortality attributable to extreme heat

We subsequently calculated the EHDs for each single heat wave observed in the 10- year interval. The expected number of excess deaths attributable to extreme heat over various lag days for the t-th heat wave was determined using Equation 4 which is referred to previous studies [24,25]:


$$\begin{array}{c} {EHD}_{ij}=\;({RR}_{ijt}-1\times {DD}_{ij}\times L_{t} \end{array}$$ (4)

Specifically, i denotes sex, j denotes age group. $L_{t}$ signifies the duration in days of the t-th phase of a heat wave. ${RR}_{ijt}$ is the relative risk of deaths for sex i, age group j, during the t-th phase of a heat wave, while ${DD}_{ij}$ is the expected number of daily deaths in the counterfactual scenario of no heat wave. We generated a distribution of 1000 random estimates of the RRs using Equation 3, then drew from the distribution to estimate ${EHD}_{ij}$ using Monte Carlo simulation. The simulated results are expressed as ${EHD}_{ijn}$**,** where n ranges from 1 to 1000, representing the Monte Carlo simulation index. This method is detailed in Zhao et al. (2024) [26].

Finally, we multiplied the cumulative count of heat waves in Hong Kong for each year from 2014 to 2023 by ${EHD}_{ij}$ to get estimates of annual excess heat-related deaths which could be added up to the total deaths by age and sex in a decade. We also reported the excess mortality as age-standardized excess death rates per 100,000 population. The age adjustment was based on the world standard population as specified in the revised age-standardization of rates by the World Health Organization – 2001 Edition [27]. The estimated excess deaths count and the age-standardized death rates across the last decade were accompanied by 95% confidence intervals (CIs). These CIs were obtained using the normal approximation with the bootstrap method described by Pizzato et al. (2024) [28], employing 1000 samples generated through Monte Carlo simulation.

We used Stata SE (v.18.0) for all statistical analyses. This study did not require ethical approval as it exclusively used publicly available data obtained from previously published. No new data was collected from human participants, and all analyses were conducted on anonymized, aggregated datasets from public sources.

## Results

### Summary statistics

During the study period in Hong Kong, average daily temperature was 24.12°C, ranging from 12°C to 31.1°C (S2 Table). As shown in Fig 1 different heat wave definitions differ in frequency and in total days of exposure over a decade. The HWD_Tcombined definition racked up the highest exposure at 365 days over 10 years, and the highest frequency of occurrences at 38 events over 10 years. The definition labeled HWD_Tavg occurred on 360 heat wave days but had the lowest frequency of 18 events. In contrast, HWD_Tmax recorded the fewest heat wave days at 183 and a frequency of 25 events. HWD_Tmin demonstrated a slightly higher frequency of 26 occurrences whereas it also had a limited duration of 190 heat wave days as illustrated in Fig 1.

**Fig 1:**
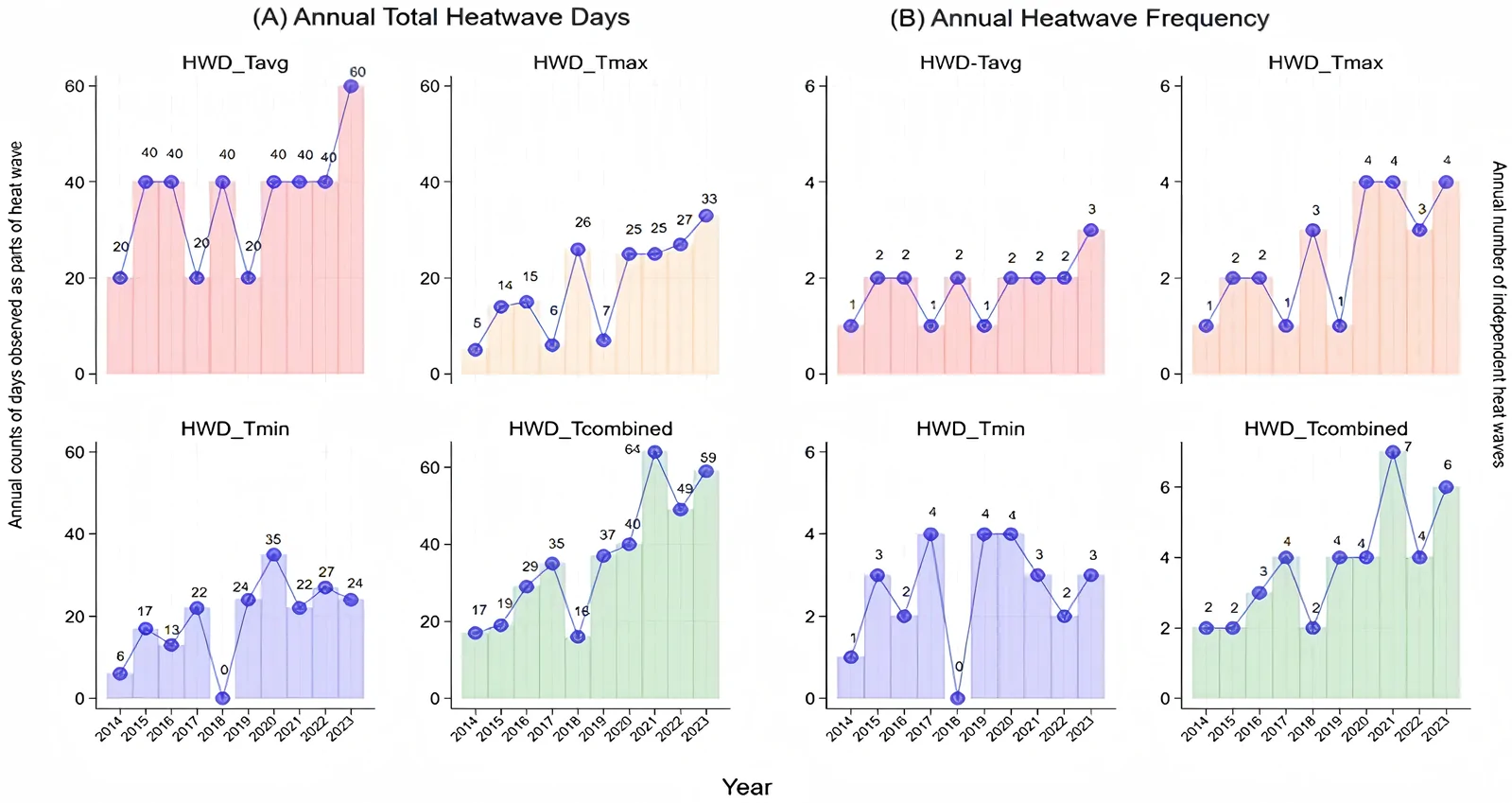
Descriptive statistics of heatwaves in Hong Kong, 2014–2023. (A) Annual sum of heatwave frequency in Hong Kong during 2014–2023. (B) Annual counts of heatwave days in Hong Kong during 2014–2023.

### Excess Mortality in number and age-standardized death rate

Based on the definition of heat event, the estimates of total excess deaths attributable to heat waves ranged from 1,455 (95% CI: 1,098–1,812) to 3,238 (95% CI: 3,234–3,242) (see Table 2 and Fig 2A). Notably, the heat wave definition based on combined temperature threshold metrics (HWD_Tcombined) resulted in more than double the estimated excess deaths compared to estimates derived from mean daily temperature (HWD_Tavg) (see Table 2). The HWD_Tavg definition gave the lowest cumulative estimates of excess heat-related deaths. See Table 2 and Fig 2A.

**Table 2:** Cumulative excess deaths attributable to heat waves over the 10-year period under different heat wave definitions.

Year	HWD_Tavg	HWD_Tmax	HWD_Tmin	HWD_Tcombined
Total	1455	2262	1553	3238
2014	82 (1098, 1812)	34 (2170, 2353)	43 (1549, 1557)	128 (3234, 3242)
2015	160 (64, 100)	97 (32, 35)	125 (41, 45)	150 (125, 131)
2016	158 (125, 196)	108 (93, 101)	100 (119, 131)	240 (147, 154)
2017	75 (121, 195)	43 (104, 113)	168 (95, 105)	280 (234, 245)
2018	147 (57, 94)	367 (41, 44)	0 (159, 176)	130 (273, 286)
2019	75 (109, 185)	103 (351, 382)	190 (0, 0)	314 (127, 133)
2020	149 (56, 94)	364 (99, 107)	278 (265, 292)	337 (306, 321)
2021	148 (110, 188)	368 (349, 379)	176 (167, 184)	538 (328, 345)
2022	202 (111, 186)	496 (353, 384)	261 (248, 274)	547 (525, 551)
2023	257 (155, 249)	282 (193, 322)	213 (203, 224)	575 (533, 560)

**Fig 2:**
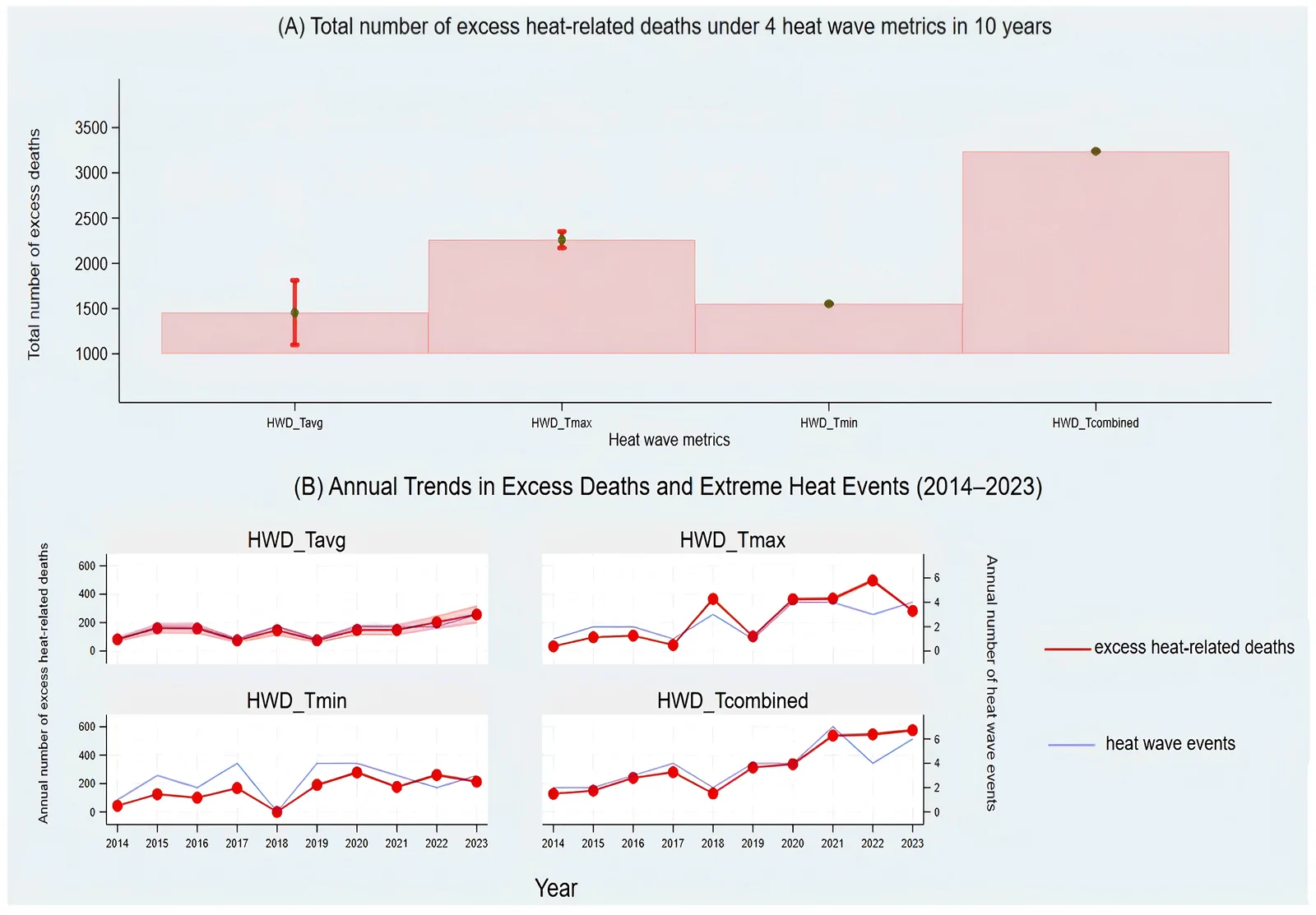
Excess heat-related deaths under four definitions, 2014–2023. (A) Total number of excess heat-related deaths across 2014–2023. (B) Annual number of excess heat-related deaths during 2014–2023.

Fig 3 presents the annual trends in excess death rates (per 100,000 individuals) in Hong Kong from 2014 to 2023 under various heat wave definitions and compares these rates to Hong Kong’s age adjusted diabetes mortality. Under the HWD_Tmax and HWD_Tcombined definitions, the excess death rate attributable to heat waves exceeded that of diabetes in nearly half of the study period (from 2020 to 2023). Notably, under the HWD_Tcombined definition, the excess death rate was nearly twice that of diabetes in the recent three years (see Table 3). Consequently, heat waves exhibiting high-risk patterns are poised to become the tenth leading cause of death in Hong Kong, even surpassing diabetes.

**Table 3:** Annual Age-standardized excess death Rate per 100,000 population in the years 2014-2023 period.

Year	HWD_Tavg	HWD_Tmax	HWD_Tmin	HWD_Tcombined	Diabetes
2014	1.18 (0.98, 1.38)	0.45 (0.44, 0.47)	0.56 (0.55, 0.56)	1.60 (1.59, 1.61)	2.5
2015	2.30 (1.89, 2.71)	1.24 (1.20, 1.29)	1.53 (1.52, 1.54)	1.80 (1.80, 1.81)	3.2
2016	2.25 (1.85, 2.65)	1.34 (1.28, 1.39)	1.19 (1.18, 1.20)	2.76 (2.75, 2.77)	3.1
2017	0.88 (0.68, 1.08)	0.44 (0.42, 0.46)	2.06 (2.05, 2.08)	2.98 (2.97, 2.99)	2.3
2018	1.74 (1.35, 2.13)	3.77 (3.61, 3.94)	0.00 (0, 0)	1.31 (1.3, 1.32)	2.8
2019	0.87 (0.69, 1.06)	1.02 (0.98, 1.07)	2.15 (2.13, 2.17)	3.07 (3.05, 3.08)	2.8
2020	1.78 (1.39, 2.16)	3.56 (3.41, 3.72)	3.12 (3.09, 3.14)	3.26 (3.24, 3.27)	3.3
2021	2.00 (1.65, 2.35)	3.53 (3.39, 3.68)	1.80 (1.79, 1.82)	5.23 (5.21, 5.25)	2.9
2022	2.46 (2.04, 2.87)	4.54 (4.35, 4.74)	2.62 (2.60, 2.64)	5.07 (5.05, 5.09)	3.5
2023	2.95 (2.41, 3.50)	2.63 (2.52, 2.75)	2.05 (2.04, 2.07)	5.09 (5.07, 5.12)	2.8

**Fig 3:**
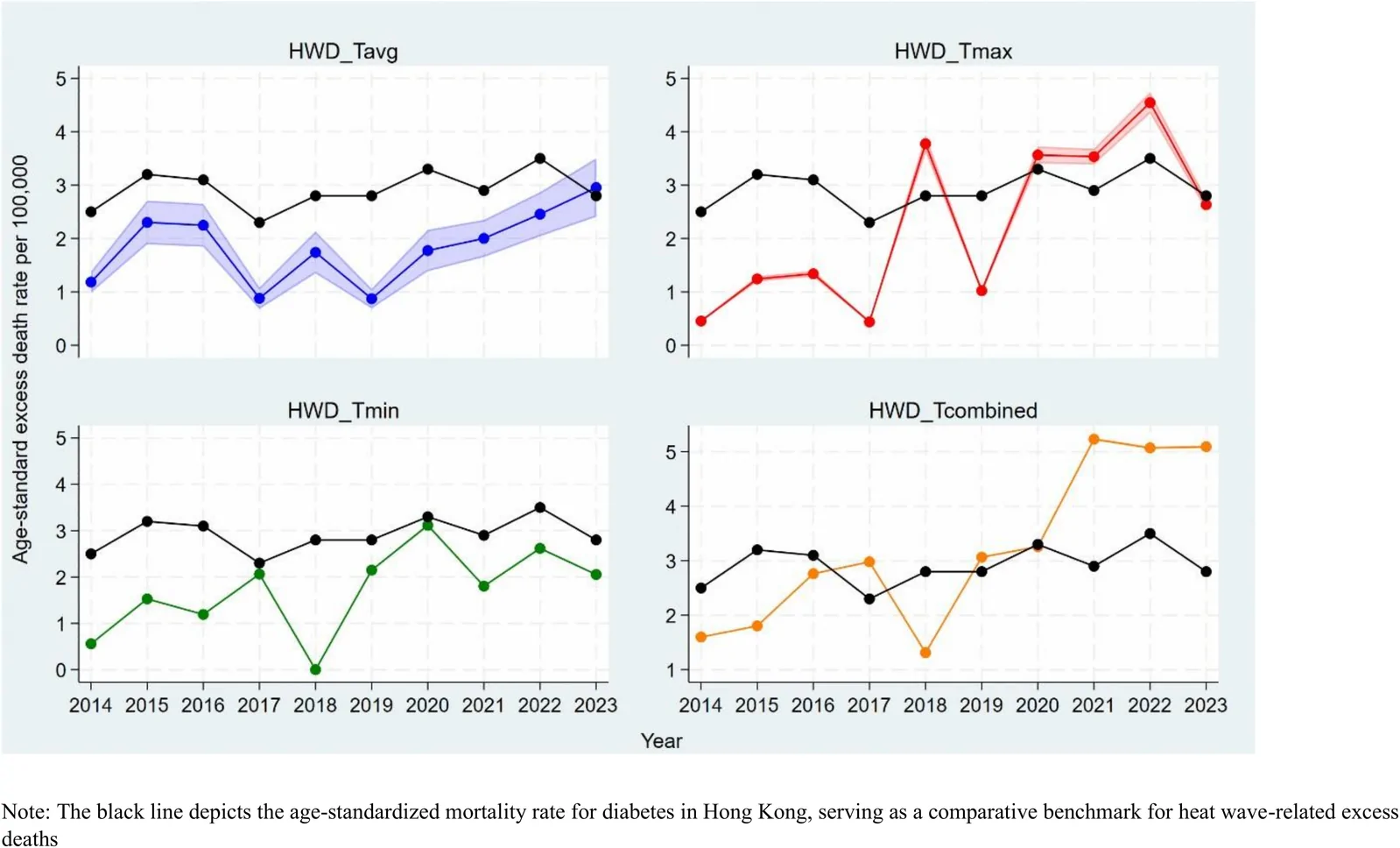
Annual trends in age-standardized excess death rates in Hong Kong, 2014–2023. Note: The black line depicts the age-standardized mortality rate for diabetes in Hong Kong, serving as a comparative benchmark for heat wave-related excess deaths.

### Sub-group analysis

Our estimate of excess death counts by age and sex reflect the epidemiological variability from the original estimates of RRs (S3 Table, S1 and S2 Figs). Males experience nearly twice the number of excess deaths compared to females across most metrics, except for the nighttime heat definition (S1 Fig). The magnitude of heat wave–related mortality risk increases with age, as individuals aged over 85 years appear to be the most vulnerable, with more than twice the number of deaths compared to other age groups across most metrics (S1 Fig). Similar demographic variability is evident in the age- standardized excess death rates (S2 Fig).

## Discussion

Overall, our estimates of excess deaths in Hong Kong under different heat wave metrics ranged from 1455 to 3238 over a 10-year period. This finding helps to compare the magnitude of risk established in previous studies to benchmark conditions like diabetes [10–12]. Notably, the magnitude of excess heat deaths shows variability across different heat wave metrics, highlighting the complexity of this public health issue. Notably, the magnitude of excess heat deaths shows variability across different heat wave metrics, highlighting the complexity of this public health issue. The substantial variability in excess death estimates across heat wave definitions requires explicit consideration. The HWD_Tcombined definition, which requires concurrent hot days at or above 33 degrees Celsius and hot nights at or above 28 degrees Celsius in a 2D3N pattern, yielded the highest mortality estimates throughout the study period. This finding is physiologically plausible, as sustained nocturnal heat suppresses the overnight thermoregulatory recovery that is essential for cardiovascular and renal homeostasis, thereby imposing cumulative physiological stress, particularly among older adults with diminished homeostatic capacity. The urban heat island effect prevalent in Hong Kong’s high density residential areas further elevates nocturnal temperatures, reinforcing the relevance of this combined threshold for the local context. In addition, the extended five-day lag incorporated into HWD_Tcombined captures delayed mortality effects that shorter interval definitions may overlook. By contrast, HWD_Tmax captures severe but potentially brief daytime heat events that may permit partial overnight recovery, whereas HWD_Tavg, which is defined by the 99th percentile mean temperature threshold with a 20-day lagged window, accounts for prolonged moderate to severe heat exposures including lagged effects up to 21 days. Despite its longer total exposure days, with 360 days over ten years compared with 183 for HWD_Tmax, HWD_Tavg produced lower estimates. This counterintuitive result reflects the fact that the relative risks reported by Liu et al. (2020) [9] are generally smaller in magnitude than those reported by Wang et al. (2019) [11] for the acute exposure phase. It is important to note that this cross-metric comparison is complicated by the fact that different source studies were used to derive the respective relative risk estimates, meaning that differences in excess death estimates reflect both the definitional characteristics of the heat wave metrics and the differing risk estimates from the underlying epidemiological studies. These two sources of variation cannot be fully disentangled within the current framework, and this should be borne in mind when interpreting cross-metric differences. These definitional differences underscore that no single heat wave metric fully captures the multidimensional physiological burden of heat exposure. Accordingly, the range of estimates generated across definitions should be considered collectively when communicating the total mortality burden to policymakers.

Furthermore, under the assumptions of this modelling exercise, and acknowledging the uncertainty inherent in applying relative risk estimates from prior studies to population level projections, extreme heat mortality, if categorized as a discrete cause of death, could be of a magnitude broadly comparable to that of diabetes. This comparison is intended to contextualize the scale of the burden rather than to assert a direct clinical or epidemiological equivalence between heat attributable modelled estimates and formally registered mortality statistics. Specifically, the excess death rate ranged from 2.95 to 5.09 per 100,000 in 2023, positioning it among the top 10 causes of death in the region [29]. It should be noted that this ranking is based on modelled estimates and is not directly equivalent to registered cause-of-death statistics, which are derived through formal clinical and administrative processes. The comparison is presented solely to illustrate the potential public health scale of heat-attributable mortality and should be interpreted with appropriate caution given the assumptions underlying the model.

Importantly, our findings reaffirm previous research indicating that individuals aged over 64 years in Hong Kong exhibit the highest susceptibility to heat wave-related excess mortality. This vulnerability is evidenced by both the excess number of deaths and the elevated death rates observed in this demographic. This well-known finding is attributed to a decreasing thermoregulatory capacity over time, as well as increased exposure to vasoactive medications for chronic diseases, which make the elderly more prone to health hazards during hot weather [30,31].

### Strengths and policy implications

To the best of our knowledge, this study is the first to estimate excess heat-related mortality in Hong Kong, encompassing both the direct and indirect impacts of extreme heat. While previous research has primarily focused on the relationship between temperature and mortality through relative risk estimates, our study shifts the emphasis to the absolute number of excess deaths, which is a more tangible and actionable metrics for policymakers and the public. Using the best available local evidence, we generated these estimates while accounting for a range of scenarios to reflect the complex and multifaceted nature of heat exposure and its health impacts.

Behavioral change could prevent excess heat deaths. Some elderly individuals in Hong Kong have shown reluctance to use effective cooling appliances to save on energy costs, even if air conditioners are installed in their homes [32]. Many flats still have poor ventilation design and small window sizes characteristic of residential buildings in Hong Kong [33]. Therefore, tailored protective measures based on population vulnerability and the specific characteristics of heat waves could prevent excess deaths. Clinicians including pharmacists, nurses, and physicians can take more initiative-taking in assessing and counseling patients about their risk from age, co-morbidities, and medications. Social agencies conducting home visits can also engage in solution finding that is in harmony with cultural beliefs.

Under global warming, Hong Kong is projected to experience more hot days and fewer cold days in the future [34], making it imperative for policymakers to develop adaptation strategies to protect vulnerable populations, especially in the context of the region’s rapidly aging population. In response to such challenges, many countries have introduced Heat Action Plans (HAPs), which include early warning systems, public advisories, and emergency health measures to reduce heat-related morbidity and mortality [35]. Empirical evidence indicates that Heat Action Plans (HAPs) can effectively mitigate the impact of extreme temperatures. For instance, studies in Ahmedabad and France reported significant reductions in all-cause mortality following HAP implementation, with Ahmedabad avoiding 2,380 deaths on heat alert days and France reporting 4,388 fewer deaths during the 2006 European Heatwave [36,37]. Similarly, a study in Montreal showed a reduction in daily mortality post-HAP, particularly among the elderly and lower socioeconomic status groups, thereby addressing heat-related health inequities [38]. Meanwhile, the transferability of these results to Hong Kong should not be assumed. Differences across jurisdictions in climate patterns, urban form, population vulnerability, healthcare capacity, housing conditions, and behavioral responses to heat may influence both implementation and effectiveness. Although Hong Kong currently does not have a dedicated HAP specifically tailored to vulnerable populations, the international evidence provides a strong rationale for considering a more integrated heat response strategy within the health and social care system. Any such strategy, however, should be informed by local epidemiological evidence, operational feasibility assessments, and pilot evaluation to ensure that interventions are contextually appropriate and effective.

### Limitations

Using local evidence can be a double-edged sword. First, in the absence of a universally accepted standard for measuring human exposure to heat, our study provides four distinct estimates of excess heat-related deaths, each based on different definitions of heatwaves used in previous local studies [9,11]. These measures can vary significantly depending on how heatwaves are characterized, such as by average temperature [9], maximum temperature, or nighttime temperature, and how deaths occurring in the days or weeks following a heatwave are accounted for [11]. Consequently, there is no single, universally accepted method for attributing deaths to heat, resulting in a range of estimates rather than a definitive value. While it remains challenging to determine which estimates are the best, we offer these as a range, inviting readers to consider the broad spectrum of possible outcomes [39]. Second, we assumed the entire territory of Hong Kong population is at equal risk of heat exposure, but heat levels vary spatially, and risk levels vary by education, occupation, socioeconomic status, and physical condition. Further stratification studies are needed to explore demographic heterogeneity.

## Conclusion

This study provides the first systematic estimates of excess deaths attributable to extreme heat in Hong Kong. Although estimates vary across heat wave definitions, reflecting the inherent complexity of attributing mortality to heat exposure, they collectively indicate a substantial and largely overlooked burden of heat related death in this densely populated subtropical city. Across all definitions examined, individuals aged 65 years and above were consistently and disproportionately affected. This finding underscores the well-established vulnerability of older adults and highlights the particular public health relevance of Hong Kong’s rapidly aging demographic profile.

These findings carry direct implications for policy, though they should be interpreted within the bounds of their underlying modelling assumptions. At the same time, the consistency of results across multiple scenarios strengthens the case for considering extreme heat as a serious and quantifiable public health threat rather than an incidental environmental hazard. Given the current absence of a formally coordinated heat response strategy in Hong Kong, the development of a territory wide HAPs represents an urgent and overdue priority. Evidence from comparable urban settings suggests that multisectoral collaboration, spanning government agencies, healthcare providers, and civil society, can produce meaningful and measurable reductions in preventable heat deaths when interventions are carefully tailored to local demographic, housing, and cultural contexts.

Under a scenario of continued warming, extreme heat is likely to constitute a growing source of preventable mortality in Hong Kong. The magnitude of this future burden will depend on three interrelated factors: climate trajectories, the adaptive capacity of the health and social care system, and the extent to which protective behaviors are embedded at the individual, household, and community levels. Proactive and coordinated investment in heat adaptation now represents the most viable pathway to limiting that burden and safeguarding the most vulnerable members of Hong Kong society.

## Supporting information

S1 Table Age-sex specific Relative risk of extreme hot temperature on mortality and sub-categories over multiple lag days in Hong Kong: under four different heat wave definitions. (DOCX)

S2 Table Descriptive statistics of daily deaths, mean temperature. (DOCX)

S3 Table Demographic heterogeneity in total number of excess heat-related deaths by age and sex. (DOCX)

S1 Fig Demographic heterogeneity in excess heat-related deaths by age and sex. (TIF)

S2 Fig Demographic heterogeneity in age-standardized mortality rates by age and sex. (TIF)
