# Supplementary material for: Modelling the excess mortality associated with heat waves in Hong Kong: 2014-2023
Source: PLoS One. 2026 Nov 13;21(11):e0345222. doi: 10.1371/journal.pone.0345222 (PMC13766615; doi:10.1371/journal.pone.0345222)
Supplement: Supplementary file 1 [file pone.0345222.s001.docx]

**S1 Table. Age-sex specific Relative risk of extreme hot**

**temperature on mortality and sub-categories over multiple lag days in Hong Kong: under four different heat wave definitions**

| **Heat wave definition 1: HWD Tavg** | | | |
| --- | --- | --- | --- |
| Lag days Female | 0 | 1-5 | 6-21 |
| 0-59 | 1.010 (0.962, 1.06) | 0.998 (0.916, 1.087) | 0.976 (0.827, 1. 15) |
| 60-74 | 0.993 (0.843, 1.046) | 1.011 (0.932, 1.097) | 1.057 (0.909, 1.228) |
| 74-85 | 1.019 (0.978, 1.062) | 1.023 (0.952, 1.099) | 1.146 (0.975, 1.347) |
| 85 +  Male | 1.022 (0.961, 1.088) | 0.976 (0.85, 1. 122) | 1.020 (0.838, 1.241) |
| 0-59 | 1.036 (0.976, 1.099) | 1.014 (0.922, 1. 115) | 0.929 (0.779, 1. 108) |
| 60-74 | 1.019 (0.957, 1.084) | 1.027 (0.937, 1. 126) | 1.006 (0.855, 1. 185) |
| 74-85 | 1.045 (0.991, 1. 103) | 1.039 (0.957, 1. 129) | 1.091 (0.917, 1.298) |
| 85 + | 1.048 (0.977, 1. 126) | 0.992 (0.858, 1. 146) | 0.972 (0.791, 1. 194) |
| **Heat wave** | **definition 2: HWD_Tmax** |  |  |
| Male |  |  |  |
| 0- 19 | 1.063 (0.835, 1.353) |  |  |
| 20-64 | 1.032 (0.938, 1. 135) |  |  |
| 65+ | 1.055 (0.983 .1.32) |  |  |
| Female |  |  |  |
| 0- 19 | 1.036 (0.812, 1.323) |  |  |
| 20-64 | 1.005 (0.908, 1. 114) |  |  |
| 65+ | 1.028 (0.949, 1. 113) |  |  |
| **Heat wave** | **definition 3: HWD_Tmin** |  |  |
| Male |  |  |  |
| 0- 19 | 1.24 (0.99, 1.55) |  |  |
| 20-64 | 1.05 (0.99, 1. 11) |  |  |
| 65+ | 1.03 (0.97, 1. 10) |  |  |
| Female |  |  |  |
| 0- 19 | 1.26 (1.00, 1.57) |  |  |
| 20-64 | 1.06 (0.99, 1. 13) |  |  |
| 65+ | 1.05 (0.97, 1. 13) |  |  |
| **Heat wave** | **definition 4: HWD_Tavg** |  |  |
| Lag days | 0 | 1-5 |  |
| Male | - | 1.038 (1.014, 1.063) |  |
| 0-19 | 0.972 (0.790, 1. 195) | - |  |
| 20-64 | 1.016 (0.926, 1. 115) | - |  |
| 65+ | 1.038 (0.968, 1. 113) | - |  |
| Female | - | 1.038 (1.014, 1.063) |  |
| 0-19 | 1.012 (0.821, 1.248) | - |  |
| 20-64 | 1.059 (0.961, 1. 167) | - |  |
| 65+ | 1.081 (1.003, 1. 166) | - |  |
