# Supplementary material for: Modelling the excess mortality associated with heat waves in Hong Kong: 2014-2023
Source: PLoS One. 2026 Nov 13;21(11):e0345222. doi: 10.1371/journal.pone.0345222 (PMC13766615; doi:10.1371/journal.pone.0345222)
Supplement: Supplementary file 2 [file pone.0345222.s002.docx]

**S2 Table. Descriptive statistics of daily deaths, mean temperature**

| **Variables** | **Mean**  **(SD)** | **Minimum** | **25th percentile** | **Median** | **75th percentile** | **Maximum** |
| --- | --- | --- | --- | --- | --- | --- |
| **Daily death counts** |  |  |  |  |  |  |
| Total | 183.82 | 164.49 | 174.13 | 181.12 | 182.54 | 224.01 |
| Gender | (16.98) |  |  |  |  |  |
| Female | 90.45  (7.74) | 80.48 | 84.85 | 89.64 | 93.87 | 106.10 |
| Male | 93.37 | 84.00 | 87.15 | 89.81 | 93.43 | 117.91 |
| Age | (9.90)  25.72 | 24.83 | 25.31 | 25.49 | 26.05 | 27.72 |
| 0-64 | (0.79) |  |  |  |  |  |
| 65-74 | 23.47  (4.01) | 25.75 | 29.07 | 32.12 | 34.31 | 42.13 |
| 75-84 | 34.94  (2.83) | 31.96 | 32.68 | 34.34 | 36.31 | 41.22 |
| 85+ | 99.69  (11.83) | 83.57 | 93.54 | 98.50 | 101.07 | 123.54 |
| **Mean** |  |  |  |  |  |  |
| **Temperature** | 24.12  (5. 10) | 12 | 20 | 25.1 | 28.8 | 31.1 |
