# Supplementary material for: Modelling the excess mortality associated with heat waves in Hong Kong: 2014-2023
Source: PLoS One. 2026 Nov 13;21(11):e0345222. doi: 10.1371/journal.pone.0345222 (PMC13766615; doi:10.1371/journal.pone.0345222)
Supplement: Supplementary file 3 [file pone.0345222.s003.docx]

**S3 Table. Demographic heterogeneity in total number of excess heat-related deaths by age and sex**

|  | **HWD_Tavg** | **HWD_Tmax** | **HWD_Tmin** | **HWD_Tcombined** |
| --- | --- | --- | --- | --- |
| **Sex** |  |  |  |  |
| Female | 0 (0, 178) | 802 (755, 849) | 877 (837, 918) | 1154 (1117, 1192) |
| Male  **Age** | 1466 (1294, 1638) | 1459 (1413, 1505) | 676 (639, 713) | 2083 (2042, 2124) |
| 0-64 | 0 (0, 0) | 241 (224, 258) | 281 (270, 291) | 382 (368, 397) |
| 65-74 | 320 (281, 360) | 342 (331, 353) | 183 (172, 193) | 481 (471, 491) |
| 75-84 | 1331 (1269, 1393) | 465 (449, 480) | 263 (249, 276) | 656 (643, 669) |
| 85+ | 0 (0, 63) | 1214 (1167, 1260) | 828 (784, 871) | 1719 (1679, 1759) |
